# The effect of folic acid or multivitamin containing folic acid supplementation during pregnancy on enamel structure of deciduous teeth: an ultrastructural and microanalytical study

**DOI:** 10.1186/s13005-025-00564-z

**Published:** 2025-11-29

**Authors:** Nermeen AbuBakr, Dina B.E. Farag, Marwa A. El-Saeed, Dina M. Elkady

**Affiliations:** 1 Oral Biology Department, Faculty of Dentistry, Cairo University, Cairo, Egypt; 2 Conservative Dentistry Department, Faculty of Dentistry, Cairo University, Cairo, Egypt

**Keywords:** Pregnancy, Folic acid, Micronutrients, Dental enamel, Preventive dentistry, Deciduous tooth

## Abstract

**Objectives:**

This work aimed to investigate the impact of folic acid or multivitamin containing folic acid supplementation during pregnancy on morphology and mineral content of enamel structure in deciduous teeth.

**Materials and methods:**

54 exfoliating deciduous upper central incisors were extracted from children between the ages of 6 and 8. Teeth were divided according to the maternal use of micronutrients into three groups (*n* = 18), group I: no use; group II: folic acid; and group III: multivitamin containing folic acid. Specimens’ ultrastructure was examined by scanning electron microscope (SEM). Elemental analysis was done using energy-dispersive X-ray spectroscopy (EDX), then statistical evaluation was conducted.

**Results:**

SEM analysis revealed a uniform enamel surface morphology, indicating a level of resistance to normal physiological enamel wear in groups II and III in contrast to group I. These findings were affirmed by EDX assessment where enamel surface revealed statistically significant higher values for calcium, and phosphorus in groups II and III relative to group I. Additionally, carbon content revealed statistically significant higher values in group I than those of groups II and III, suggesting more susceptibility of hydroxyapatite crystals to dissolution in group Ι.

**Conclusion:**

Maternal micronutrients supplementation was linked with the preservation of enamel structure in primary teeth. This indicates enhanced enamel resistance to mineral loss during normal physiological dental wear. The findings of this preliminary study highlighted the importance of prenatal multivitamin supplementation as a strategy in preventive dentistry.

**Supplementary Information:**

The online version contains supplementary material available at 10.1186/s13005-025-00564-z.

## Introduction

Dental development is a dynamic and ongoing process influenced by epithelial-mesenchymal interaction. It is also affected by environmental, epigenetic and genetic variables across life [1]. As an environmental factor, several micronutrients can help regulate dental growth [2]. The first evidence of tooth development is thickened oral epithelium on the 11th day of pregnancy, followed by permanent dentition in week 20 of gestation [3]. Thus, micronutrient deficits at any of these critical time points can directly impact tooth development and mineralization as well as matrix secretion in hard dental tissues [4].

Vitamins, as necessary micronutrients, assist a variety of human body biological processes. They are also necessary for children’s stomatognathic system to grow and develop in a healthy manner. According to their solubility, vitamins are distinguished into two groups: water-soluble vitamins (C and B), which are necessary for enzyme activation, and fat-soluble vitamins (A, D, E, and K), that are vital for the fluidity of cell membranes [5, 6].

Vitamin imbalance can result in malnutrition. Malnutrition during development can impact the formation of oral structures as well as the advancement of oral illnesses due to decreased resistance to microbial biofilms, altered tissue homeostasis, and impaired tissue healing capacity [7]. Vitamin deficiency throughout pregnancy and early infancy has a significant impact on tooth mineralization and development. There is evidence linking deficiencies in vitamin D, C, B, and A to oral structural abnormalities. It was hypothesized that enamel hypoplasia and caries in primary dentition were positively correlated with malnutrition in children [8, 9].

Of these vitamins, folic acid, sometimes referred to as vitamin B9 or folacin, and the naturally occurring form, folate, are water-soluble B-complex vitamins that are required for the synthesis and replication of cellular genetic material [10]. Folic acid is particularly important for oral health since it prevents the development of many diseases of the teeth, oral mucosa and periodontium, along with congenital defects in the oro-maxillo-facial area, such as orofacial clefts and neural tube defects [11]. Vitamin B12 and B9 insufficiency in children has been linked with higher prevalence of caries and gingival issues [12].

Since diet only provides trace amounts of these essential nutrients, maternal multiple micronutrient supplementation (MMS) is advised to maintain both the mother’s health and the developing fetus and to prevent issues related to micronutrient deficiencies during pregnancy [13]. The scientific data highlighting the influence of different prenatal micronutrient supplements’ intake on dental development and mineralization in children is limited. Therefore, the aim of the current investigation was to examine the impact of prenatal folic acid or multivitamin containing folic acid on the enamel structure of deciduous teeth. These effects were evaluated ultrastructurally and microanalytically. The null hypothesis of this study is that prenatal micronutrients supplementation would not impact the enamel structure of deciduous teeth.

## Materials and methods

### Sample size calculation

To apply a statistical test of the null hypothesis, a power analysis was created. In accordance with the findings of a prior investigation [14], an effect size (f) of 0.555 and alpha (α) and beta (β) levels of 0.05 (i.e., power = 95%) were adopted. It was determined that 54 samples, (i.e., 18 samples for each group), were the total necessary sample size (n). To calculate the sample size, R statistical analysis software (version 4.3.2 for Windows) was used.

### Selection of subjects (mother-child pairs)

Data on mother-child pairs were collected using a structured, standardized questionnaire (see supplementary material).

#### Mothers

Maternal history of vitamin intake was obtained from mothers who had documented prescriptions for vitamin supplementation during pregnancy (self-reported intake without a prescription was excluded). Only mothers who took 0.4 mg of folic acid once daily throughout pregnancy or multivitamin tablets, including a combination of essential vitamins and minerals like vitamins D, C, A, E, K, B-complex, iron, calcium, and 0.4 mg of folic acid once daily during pregnancy, were included in the study. A proper case history for the pregnancy period was taken from the mother to ensure being free from any systemic diseases such as hypertension, autoimmune diseases or diabetes that may affect maternal and fetal nutrition or gastrointestinal or metabolic conditions that may impact the absorption of nutrients such as malabsorption syndromes or celiac disease.

#### Children

The children recruited for the study were randomly selected, visiting the Pedodontics department, Cairo University’s Faculty of Dentistry to undergo deciduous upper central incisor tooth extraction due to either pre-shedding hypermobility or retention. Children born to mothers who fulfilled the maternal inclusion criteria for vitamin supplementation were included in this study. To ensure a homogenous study population and reduce confounding variables, inclusion criteria were adopted. Only children aged 6–8 years, with no systemic diseases or medical issues (such as genetic or metabolic disorders) that can impact enamel development were included in the study. Additionally, the included children had good oral hygiene, defined as brushing once- twice a day with fluoride toothpaste, as stated by parents and verified by clinical examination, and had a DMF (decayed-missing-filled) score of ≤ 2 indicating a low caries risk. Moreover, they had low sugar intake defined as consuming sugary snacks and beverages 3–5 times a week and had balanced nutrition including fruits, vegetables, proteins and dairy products. Furthermore, they use fluoridated toothpaste and drink fluoridated tap water but were not previously subjected to professional preventive measures such as topical fluoride application.

### Specimen Preparation

After extraction, the teeth were washed of soft tissue debris and blood and then disinfected. Teeth were then kept in a refrigerator at 4 ± 0.1 °C for no more than a week in deionized water [15].

### Study design

The study comprised 54 teeth which were split into three groups (18 teeth each), based on whether the mothers administered folic acid alone or in conjunction with a multivitamin, as follows: Group I: No use, identified as no usage of folic acid or multivitamin; Group II: Folic acid, identified as folic acid intake throughout the whole period of pregnancy; and Group III: Multivitamin, defined as multivitamin containing folic acid intake throughout the whole pregnancy. The exfoliating teeth obtained were subjected to the following procedures:

### Scanning electron microscopy (SEM)

SEM examination was carried out to analyze the enamel’s surface morphology. Before imaging, the tooth sample was properly washed in deionized water, then air-dried. The mid of the inscisomiddle 2/3 of the crown labial portion was the scanning site, which was standardized for all tooth samples. All the specimens were examined under environmental SEM (Quanta 250 FEG (field emission gun)/EDS, Octane Pro, USA).

### Energy-dispersive X-ray spectroscopy (EDX)

Following SEM imaging, elemental analysis of enamel surface in all groups was carried out in the same areas of the crown. The EDX analysis system functions as an integral component of the SEM Quanta FEG 250. Calcium (Ca), Phosphorus (P) and Carbon (C) weight% was assessed.

### Statistical analysis

The statistical program SPSS version 22 was used to code and enter numerical data (IBM Corp., Armonk, NY, USA). By examining the data distribution and applying the Kolmogrov-Smirnov test, the data, which were displayed as mean ± standard deviation, were examined for normality. The information was dispersed normally. The data was then analyzed using one-way ANOVA, with numerous pairwise comparisons done using Tukey’s post hoc test. A P-value of 0.05 or less was judged statistically significant.

## Results

### SEM results

#### Group I

**Fig. 1:**
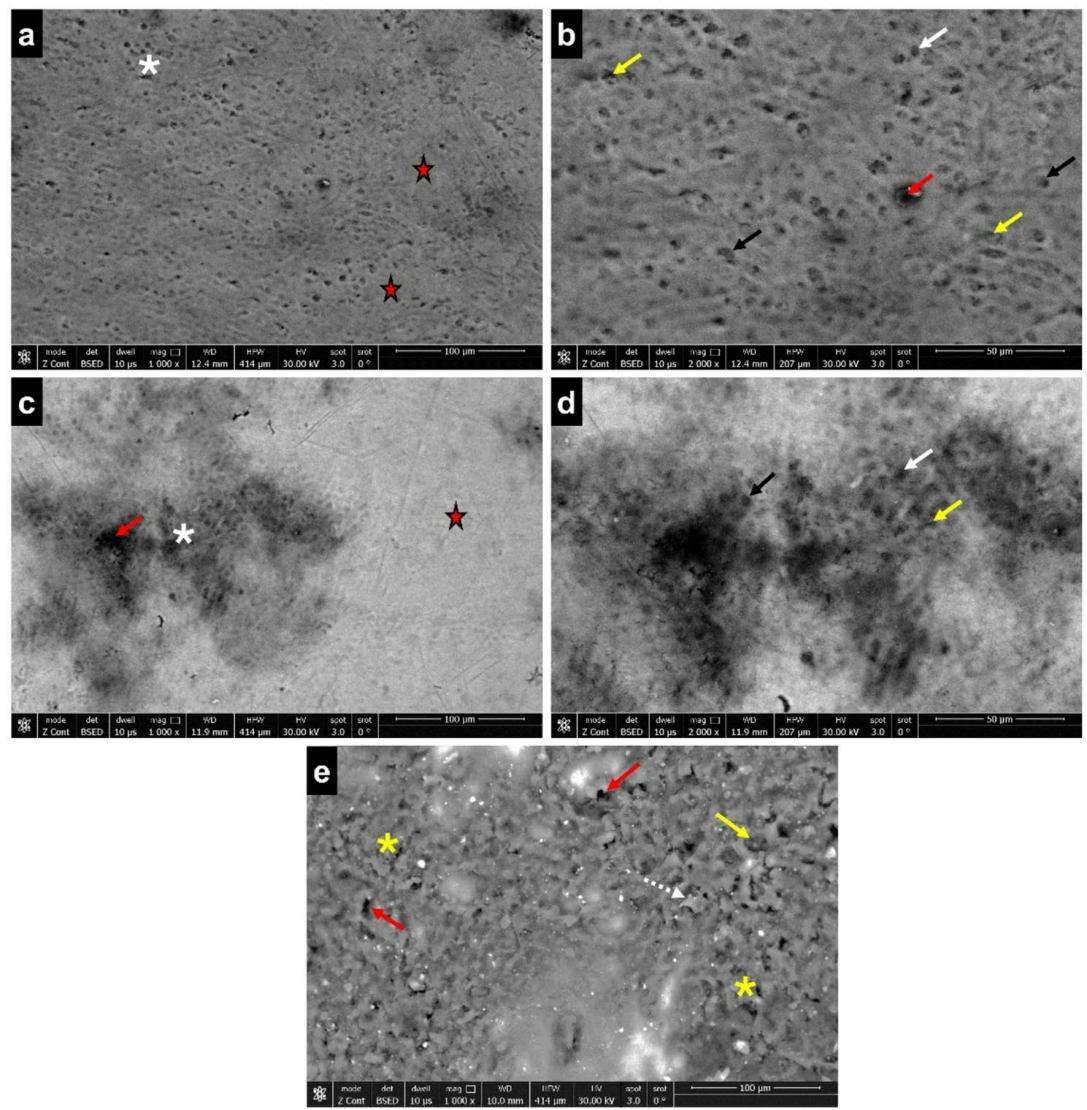
SEM image of enamel surface (group I) showing; **(a-e)** exposed enamel rod areas (white asterisks), surface rodless enamel (red stars), microporosities (red arrows), loss of enamel rod core (white arrows), partial dissolution of enamel rod core (yellow arrows), intact rod peripheries (black arrows), rough enamel surface (yellow asterisks), sharp enamel projections (white dotted arrow) (a, c, e; scale bar = 100 μm, b, d; scale bar = 50 μm)

The SEM images of enamel in group I showed an inhomogeneous surface. Various morphological patterns of the enamel surface were noted in the specimens under examination. Most of the specimens displayed intermingling between areas of surface rodless enamel and areas of exposed enamel rods. The exposed enamel rods exhibited varying degrees of dissolution; some specimens showed full dissolution of the enamel rod core, while others only exhibited partial dissolution. However, most of the rod peripheries were intact **(**Fig. 1a-d**)**. The enamel surface in other samples appeared severely roughened with scattered microporosities and some sharp enamel projections. Minimal enamel rods exposure was noted **(**Fig. 1e**)**.

#### Group II

**Fig. 2:**
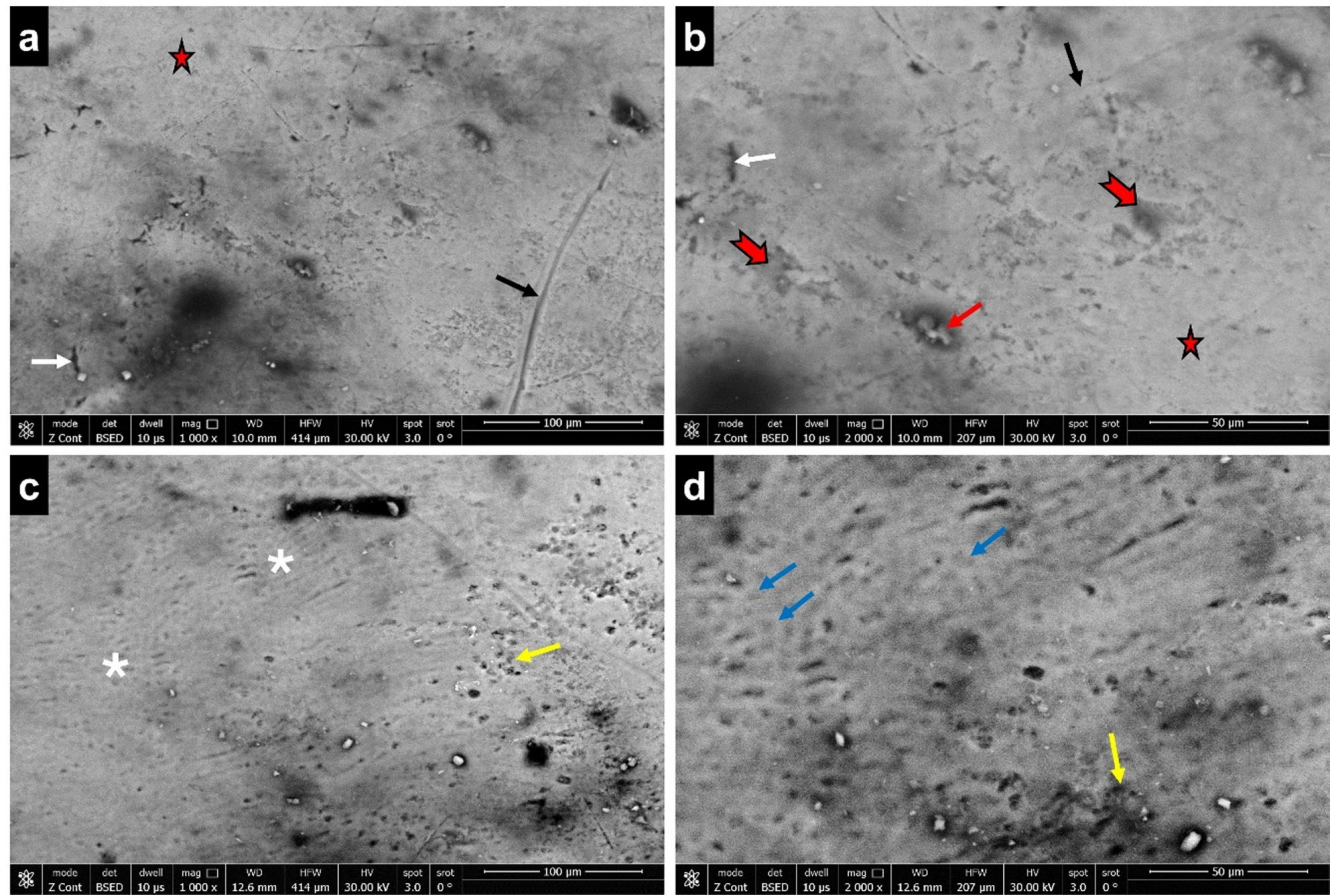
SEM image of enamel surface (group II) showing; **(a-d)** surface rodless enamel (red stars), multiple scratches (black arrows), irregular patches (notched red arrows), fine surface cracks (white arrows), microporosities with globular particles (red arrow), enamel rod exposure (white asterisks), preserved rod core and periphery (blue arrows), numerous micropores (yellow arrows) (a, c; scale bar = 100 μm, b, d; scale bar = 50 μm)

Compared to group I, the enamel surface in group II had less surface defects and was partially smooth. Surface rodless enamel was evident in most of the specimens. Multiple scratches, uneven irregular patches, fine surface cracks, and dispersed microporosities with or without globular particles were also noted. There were exposed enamel rod areas, but the majority of the rods’ core and periphery were still intact (Fig. 2).

#### Group III

**Fig. 3:**
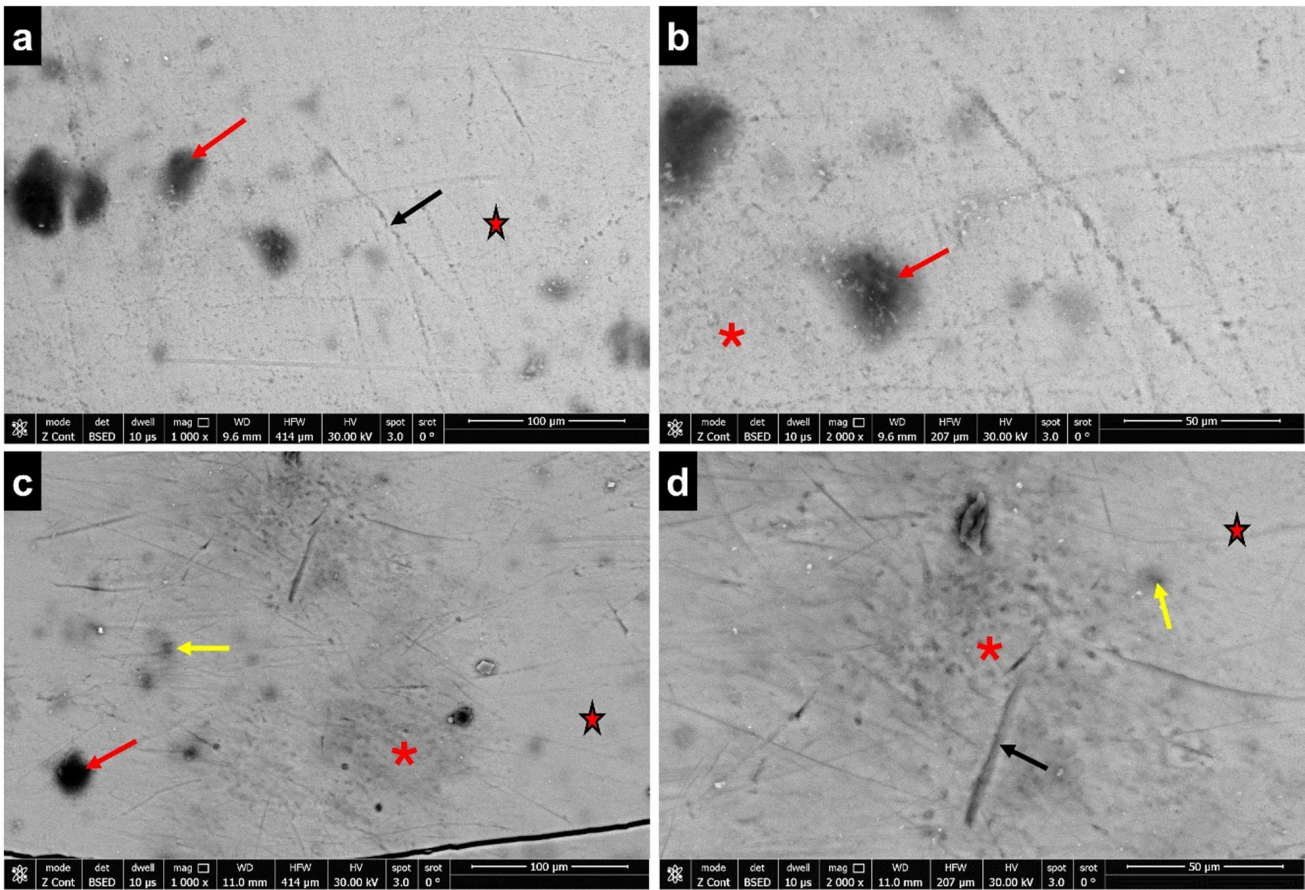
SEM image of enamel surface (group III) showing; **(a-d)** surface rodless enamel (red stars), surface scratches (black arrows), holes of different sizes (red arrows), rough areas (red asterisks), micropores (yellow arrows) (a, c; scale bar = 100 μm, b, d; scale bar = 50 μm)

The enamel surface in group III showed a more uniform and smoother surface when compared to groups I and II. In the majority of the specimens, the surface rodless enamel remained intact with no discernible exposure to enamel rods. Microporosities, sporadic rough patches, and occasional scratch marks were all visible. Holes of different sizes also characterized the surface (Fig. 3).

### Energy dispersive X-ray (EDX) results

#### Ca results

**Fig. 4:**
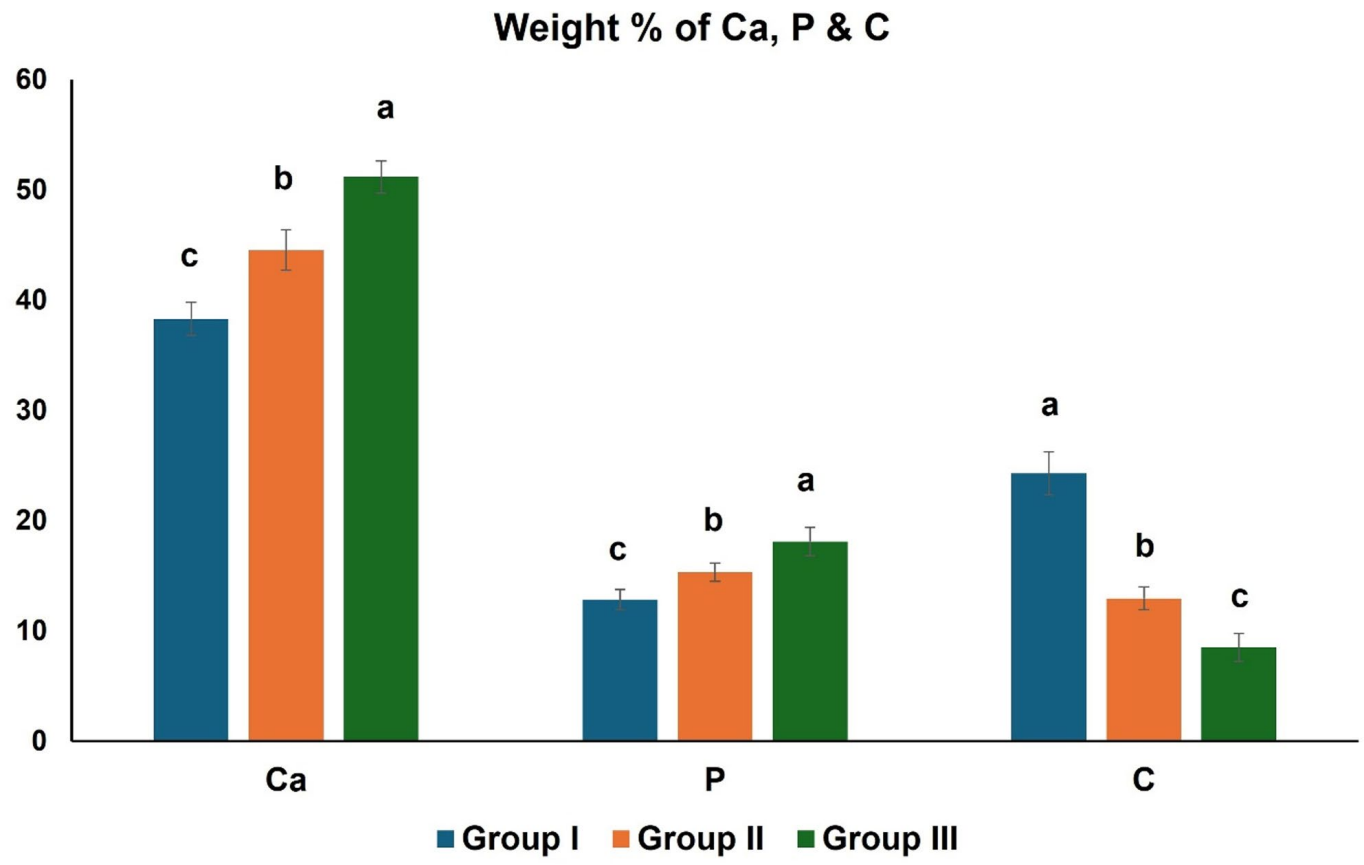
Bar chart displaying mean and standard deviation values of weight% of Ca, P & C. Bars with different letters are significantly different

The greatest Ca content was detected in group III (51.17 ± 1.45), while the lowest content was seen in group I (38.29 ± 1.48). The variation between all groups was determined to be of statistical significance (*p* < 0.001). Further, several pairwise comparisons among the groups showed that the increase in Ca in groups II and III was statistically significant with respect to group I (*p* < 0.001). There was also a statistically significant elevation in group III compared to group II (*p* < 0.001) **(**Fig. 4**)**.

#### P results

The highest P content was demonstrated in group III (18.09 ± 1.29), while group I showed the lowest values (12.80 ± 0.92). The distinction between all groups was significant statistically (*p* < 0.001). Both groups II and III had a significantly elevated P content than group I (*p* = 0.006 and *p* < 0.001, respectively). Additionally, P content was significantly elevated in group III in comparison to group II (*p* = 0.003) **(**Fig. 4**)**.

####  C results

The difference in C content across groups was significant (*p* < 0.001), with the greatest values seen in group I (24.27 ± 1.94), and the lowest values observed in group III (8.49 ± 1.26). The C content in groups II and III was declined significantly compared to group I (*p* < 0.001). Additionally, a statistical decrease in C was detected in group III in comparison to group II (*p* = 0.001) **(**Fig. 4**)**.

## Discussion

Maternal nutrition is crucial not only for supplying the basic nutrients required for fetal growth, but it also controls fetal gene expression patterns that determine the patterns of physiological and metabolic reactions, and future illness risk [16]. MMS has been included in the WHO’s Model Essential Medicines List as a prenatal supplement for pregnant women as of October 2021 [17]. This work attempted to investigate the impact of MMS on enamel structure in primary teeth by assessing surface morphological alterations and mineral content. In the current work, we demonstrated that the enamel structure integrity was more preserved in deciduous central incisors obtained from children whose mothers received folic acid only or received multivitamin containing folic acid during pregnancy as compared to those obtained from children whose mothers did not take any micronutrient supplements during pregnancy.

Deciduous teeth are distinct biological tissues as they permanently record both prenatal and early postnatal conditions. Primary tooth buds start from the embryonic stage and continue to develop and mineralize during pregnancy, their mineralization and development can be affected by the intrauterine conditions, especially the nutritional state of the mother [18].

Human deciduous central maxillary incisors are the first teeth to start calcification, which occurs approximately between the 13th and 15th weeks of pregnancy [19–21]. It had been reported that, at birth, most of these tooth crowns are formed (approximately three fifths), and about of five sixths of enamel is formed [22, 23], and thus these teeth may provide a record of the environment throughout pregnancy. Accordingly, the deciduous central incisor teeth were selected as an appropriate tooth type for this work, and the inscisomiddle 2/3 served as the examination area representing the prenatal enamel [22].

In this study, the SEM images of enamel surface from group I (no MMS) displayed regions where enamel rods were exposed. Additionally, apparent surface roughness was demonstrated. The herein observed surface morphology suggested that there was partial dissolution of the enamel surface, which might have arisen from different factors, including attrition, abrasion, and erosion due to the natural wear of enamel, a common occurrence during the period of deciduous dentition. This was consistent with SEM results reported in an earlier investigation, which demonstrated mild demineralization and a roughened, scratched enamel surface in primary teeth subjected to wear challenge using oral wear simulator machine [24]. Since primary tooth enamel is weaker and thinner than that of permanent teeth, it has been proposed that tooth wear may occur more quickly in primary teeth [25].

In contrast to group I, ultrastructure analysis for groups II (maternal folic acid) and III (maternal multivitamin containing folic acid) in this study, displayed noticeably smoother enamel surface morphology, retaining much of the surface rodless enamel. These findings suggested a greater likelihood of a sound enamel surface, reflecting a degree of resistance to normal physiological enamel wear in these experimental groups when compared to group I.

Assessing enamel mineral content could serve as an indicator of environmental exposure to various elements throughout enamel development and maturation phases [26]. Quantitative evaluation for mineral changes on the enamel surface in this study using EDX revealed higher significant mean values for Ca, and P in groups II and III relative to group I. Since the main constituents of enamel hydroxyapatite are Ca and P, higher concentrations of these ions indicated a greater degree of enamel mineralization [25]. Going through this work’s EDX results, C content revealed statistically significant higher values in group I as compared to both groups II and III. According to a report by Alkattan et al. [27], C ions can replace phosphate ions and at high concentrations, hydroxyl ions as well, making the crystal less stable and exacerbating the dissolution of apatite. Given the current study’s elemental analysis, it could be inferred that group I’s substantially higher C content and lower Ca, and P content indicates that the hydroxyapatite crystals were more susceptible to dissolution than those in groups II and III.

In analyzing the SEM and EDX findings of this study, it was noted that the surface structure of enamel in group III was more uniform, exhibiting significantly higher content of Ca and P and lower content of C compared to group II. This suggests that maternal folic acid supplementation along with multivitamin could lead to more positive biological effects on enamel-forming cells, subsequently influencing the integrity of the enamel structure, compared to maternal folic acid supplementation alone. This assumption was based on the fact that vitamins and trace elements are necessary for several metabolic processes that employ carbohydrates, fats and proteins for growth, energy production, and cellular maintenance [28].

The exact biological mechanism through which maternal folic acid and multivitamin supplementation preserved the integrity of enamel structure observed in this study is not fully understood, given that research focusing on the impact of MMS during pregnancy on dental development overall and specifically on enamel formation and mineralization is few [29–31].

In regard to folic acid, it facilitates the transfer of methyl groups between molecules, which is vital for amino acid metabolism and nucleotide synthesis; thus, it is necessary for cell proliferation, differentiation, and maintenance of new cell formation [32].

In a cohort study, inadequate folic acid intake throughout pregnancy raised the risk of early childhood caries in young children. This research identifies folic acid deficiency as a contributing factor to the onset of early childhood caries [33]. Folic acid insufficiency was shown to be directly engaged in the cariogenic process by raising the levels of oxidative stress indicators in saliva [34].

Although the reported protective effect of folic acid against tooth decay [35] aligned with the results of this work, another research by Dhamo et al. [29] found a link between maternal preconception and post conception folic acid intake and delayed tooth development in children. As a hypothetical explanation for this observation, the authors suggested that folic acid could be involved in stimulating tooth mineralization inhibitors like pyrophosphate. These outcomes were not in line with our findings.

Vitamins D, C, E, B12 and B6, are the most reported active ingredients in popular maternal supplements [36]. Vitamin D is important for controlling the levels of phosphorus and calcium in the blood, aiding in their absorption in the intestine and reabsorption in the kidney [37, 38]. The fetal teeth’s developmental phases rely on vitamin D, indicating that prenatal vitamin D levels throughout pregnancy will influence tooth calcification. 1,25-dihydroxy vitamin D3, an active form of 25(OH)D, appears to function by upregulating the vitamin D receptor, which may result in the production of structural gene products like calcium-binding proteins and various extracellular matrix proteins (including amelogenins and enamelins), which in turn cause the formation of enamel [39–41]. Supplementation with vitamin D throughout pregnancy has been linked with a decreased risk of enamel abnormalities in neonates, highlighting its protective function against enamel insufficiency [30]. According to Beckett et al. [31], a significantly increased risk of caries in the deciduous dentition by the age of six was linked to vitamin D insufficiency during the third trimester of pregnancy.

A crucial antioxidant, vitamin C, participates in various enzymatic activities, including those that produce neuropeptides, carnitine, and collagen [42]. Vitamin C supports the strength of bones and teeth during pregnancy by aiding in collagen growth and repair [43]. In the development of tooth structure, vitamin C was among other vitamins that were required for appropriate calcium deposition and calcification [28]. Earlier work by Amar et al. [44] observed that vitamin C deficiency hampered the functional differentiation of ameloblasts in mouse incisor tooth germs in vitro.

Vitamin B12 is an essential micronutrient for cellular formation and metabolism as well as the formation of both myelin and DNA. In a previous study involving 3,728 moms and their kids, researchers examined the relationships between maternal vitamin B12 consumption and the dental development of children at 10 years old. It was noted that elevated maternal vitamin B12 levels during the first trimester of pregnancy were linked to faster dental development [29].

Pre-formed vitamin A (retinol) or beta carotene were noted to be present in approximately 35% and 73% of prenatal supplements respectively [45]. Vitamin A is essential for maintaining the differentiation and integrity of epithelial cells. It has been indicated that vitamin A influences ameloblast function during the process of enamel development [28, 46]. Multiple reports indicated that a lack of vitamin A might weaken the integrity of enamel structure [47, 48].

Besides vitamins, prenatal micronutrient supplements contained several essential minerals. Essential minerals and trace elements were observed to have an important role in teeth development, including dentinogenesis and amelogenesis [49]. Calcium is one of the most frequently found minerals in MMS [50]. It is vital to the mineralized and ordered crystalline structure of enamel, as it interacts with inorganic phosphate to generate hydroxyapatite and is involved in ion transport throughout amelogenesis. It was reported that low maternal calcium levels were substantially linked to enamel hypoplasia [51, 52].

Finally, some limitations need to be addressed regarding this work. First, this research was conducted with a small sample group and provided only an objective observation, where the outcomes should be considered preliminary. Second, although we attempted to discuss the potential actions of the main components in maternal multivitamin supplements in relation to enamel formation and mineralization, the complexity of multivitamin composition did not allow determination of which specific vitamin or mineral used in the formulation contributes to the preservation of enamel structure integrity observed in this study.

## Conclusion

According to the findings acquired from this work, the consumption of folic acid or multivitamin containing folic acid during pregnancy was linked with maintenance of enamel structure integrity in primary teeth. This indicated enhanced enamel resistance to mineral loss during normal physiological dental wear. However, more studies are needed to provide stronger evidence for this outcome. Employing animal models to identify the molecular mechanisms via which various maternal micronutrients components and levels regulate enamel development, particularly studying ameloblasts during the secretory and maturation stages of amelogenesis is required. Future large-scale clinical trials that include biochemical assessment of maternal vitamin levels and short- and long-term dental follow-up for children may enable researchers to validate the importance of prenatal multivitamin supplementations as a strategy in preventive dentistry.

## Supplementary Information

Supplementary Material 1.

## Data Availability

All data generated or analyzed during this study are included in this published article.

## Abbreviations

Ca: Calcium
C: Carbon
EDX: Energy dispersive X-ray
MMS: Multiple micronutrient supplementation
P: Phosphorus
SEM: Scanning electron microscope
