# Supplementary material for: The effect of folic acid or multivitamin containing folic acid supplementation during pregnancy on enamel structure of deciduous teeth: an ultrastructural and microanalytical study
Source: Head Face Med. 2025 Nov 29;21:85. doi: 10.1186/s13005-025-00564-z (PMC12702154; doi:10.1186/s13005-025-00564-z)
Supplement: Supplementary file 1 — Supplementary Material 1. [file 13005_2025_564_MOESM1_ESM.docx]

**Maternal Vitamin Intake Questionnaire**

[To assess maternal vitamin supplementation during pregnancy]

**Section 1: General Information**

1. Did you have any diagnosed medical conditions during pregnancy? (any systemic diseases during pregnancy (diabetes, hypertension, autoimmune disease)?

☐ Yes (If yes, please specify: ___________) [Not eligible]

☐ No

2. Do you have any metabolic or gastrointestinal disorders affecting nutrient absorption?

☐ Yes (If yes, please specify: ___________) [Not eligible]

☐ No

**Section 2: Vitamin Supplementation During Pregnancy**

3. Did you take prescribed prenatal vitamins during pregnancy?

☐ Yes (Please specify: ___________)

☐ No

4. Can you provide documentation of your vitamin prescription (prescription records, doctor’s notes)?

☐ Yes

☐ No [Not eligible]

5. During which trimester did you take these vitamins?

☐ First trimester [Not eligible]

☐ Second trimester [Not eligible]

☐ Third trimester [Not eligible]

☐ Throughout pregnancy

**Child Inclusion Criteria Questionnaire**

**Section 1: General Information**

1. Child’s age: ____ years (Only children aged 6-8 years are eligible).

2. Has your child been diagnosed with any medical conditions affecting their general health?

☐ Yes (If yes, please specify: ___________) [Not eligible]

☐ No

**Section 2: Oral Hygiene Practice**

3. How often does your child brush their teeth?

☐ Twice daily or more (Low risk)

☐ Once daily (Moderate risk)

☐ Less than once daily [Not eligible]

4. Does your child use fluoride toothpaste?

☐ Yes

☐ No [Not eligible]

**Section 3: Caries Index (Clinical Examination)**

5. DMF (Decayed-Missing- Filled) Score (To be completed by examiner): ____

(Eligible if dmft score is ≤2, as recommended by caries risk assessment tools)

**Section 4: Dietary Habits**

6. How often does your child consume sugary snacks and drinks?

☐ Rarely (Less than 3 times per week) [Low risk]

☐ Occasionally (3-5 times per week) [Moderate risk]

☐ Frequently (More than 5 times per week) [Not eligible]

7. Does your child follow a balanced diet including fruits, vegetables, proteins, and dairy products?

☐ Yes

☐ No [Not eligible]

**Section 5: Fluoride Exposure**

8. What is your primary source of drinking water?

☐ Fluoridated tap water

☐ Non-fluoridated water (e.g., well water) [Not eligible]

9. Has your child received professional fluoride treatments or supplements?

☐ Yes [Not eligible]

☐ No
